# Quality Evaluation and Antioxidant Activity of Cultivated *Gentiana siphonantha*: An Ethnic Medicine from the Tibetan Plateau

**DOI:** 10.3390/molecules31020312

**Published:** 2026-01-16

**Authors:** Jiamin Li, Liyan Zang, Xiaoming Song, Zixuan Liu, Hongmei Li, Jing Sun

**Affiliations:** 1 College of Pharmacy, Qinghai Minzu University, Xining 810007, China; 19953109651@163.com (J.L.); xiaomingwk925@163.com (X.S.); 2 Qinghai Provincial Key Laboratory of Qinghai-Tibet Plateau Biological Resources, Northwest Institute of Plateau Biology, Chinese Academy of Sciences, Xining 810008, China; zangliyan@nwipb.cas.cn (L.Z.); 18562277650@163.com (Z.L.); lihongmei@nwipb.cas.cn (H.L.); 3 University of Chinese Academy of Sciences, Beijing 100049, China; 4 School of Life Sciences, Yantai University, Yantai 264005, China

**Keywords:** *Gentiana siphonantha*, iridoid glycosides, quality evaluation, antioxidant activity

## Abstract

*Gentiana* species are widely used in traditional and modern medicine, and *Gentiana siphonantha* is an important medicinal representative. To evaluate the quality characteristics of cultivated *G. siphonantha* roots, the accumulation patterns of iridoid glycosides and antioxidant activities across different cultivation ages and harvest months were investigated. Five major iridoid glycosides were quantified, and antioxidant capacity was assessed through DPPH, ABTS, and FRAP assays. Quality was subsequently multidimensionally evaluated using principal component analysis (PCA), orthogonal partial least squares–discriminant analysis (OPLS-DA), membership function analysis, and entropy weight–TOPSIS analysis, and the relationship between iridoid glycoside content and antioxidant activity was analyzed. Results showed that 3-year-old cultivated roots had the highest total iridoid glycoside content (134.60 mg·g^−1^ DW), indicating the optimal cultivation age. Peak glycoside accumulation occurred in 4-year-old plants harvested in June–July, identifying this period as the optimal harvest time, as supported by multivariate statistical and comprehensive evaluation. Antioxidant activity increased with cultivation age, with samples collected in June or August showing higher capacities, and it was positively correlated with total iridoid glycoside content, particularly with FRAP (*p* < 0.05). In conclusion, cultivated *G. siphonantha* exhibits stable quality and favorable antioxidant activity, providing a basis for standardized cultivation, quality evaluation, and rational utilization.

## 1. Introduction

*Gentiana siphonantha* Maxim. ex Kusn. (*G. siphonantha*), a perennial herbaceous plant in the genus *Gentiana* of the family Gentianaceae, is used medicinally for its roots. It serves as an independent Qinjiao-type medicinal material within Tibetan, Mongolian, and other traditional medicine systems and possesses significant medicinal values [1]. Current research on *G. siphonantha* mainly focuses on pharmacognosy [2], ecology [3], chemical constituents [4], and molecular biology [5]. For example, previous phytochemical studies on *G. siphonantha* root have identified two new acylated secoiridoid glycosides and five known secoiridoids, including gentiolactone, gentiopicroside, sweroside, gelidoside, and trifloroside, and elucidated their structures using NMR techniques [6]. Their findings also demonstrated the close relationship of iridoid glycoside profiles between *G. siphonantha* and *G. macrophylla*, supporting the use of *G. siphonantha* as a potential substitute for *G. macrophylla*. Additionally, previous scholars [7] developed a precise and sensitive LC/UV method for the simultaneous determination of four iridoid and secoiridoid glycosides (loganic acid, swertiamarin, gentiopicroside, and sweroside) in the roots of six *Gentiana* species, including *G. siphonantha*. This study represented the first validated LC fingerprint comparison and quantitative evaluation of multiple genuine *Gentiana* species and their substitutes. This finding further suggests that *G. siphonantha* could serve as a reasonable substitute for *G. macrophylla*. The plant contains active compounds such as iridoid glycosides, flavonoids, and phenolic acids, exhibiting multiple pharmacological activities, including anti-inflammatory, antioxidant, hepatoprotective, and immunomodulatory effects [2,4]. Previous studies have shown that the in vitro antioxidant activity of extracts from the roots of various *Gentiana* species is mainly related to their phenolic components [8]. Moreover, recent studies have indicated that iridoid glycosides also exhibit antioxidant activity. For instance, swertiamarin, a representative iridoid glycoside, has been reported to exert anti-inflammatory effects in diabetic rats and display antioxidant activity [9,10]. These findings suggest that iridoid glycosides are not merely bioactive but may also participate in oxidative stress regulation. In addition, several studies [11,12,13,14] have shown that other iridoid glycosides, particularly gentiopicroside, exhibit antioxidant potential through mechanisms such as free-radical scavenging and modulation of oxidative stress-related pathways. Taken together, these studies indicate that iridoid glycosides may play a meaningful role in the overall antioxidant activity observed in our samples and may complement the contribution of phenolic constituents. Considering that iridoid glycosides have been reported to exhibit antioxidant activity, and that gentiopicroside and loganic acid are designated marker compounds in the 2025 edition of the Pharmacopoeia of the People’s Republic of China (ChP) [15] and are also commonly used as characteristic constituents in traditional ethnomedicine research [16], iridoid glycosides were selected as the research targets of this study. Therefore, it is essential to further investigate the association between these compounds and antioxidant activity.

Furthermore, wild *G. siphonantha* resources are primarily distributed in high-altitude, cold, and arid regions of Qinghai, Sichuan, and Ningxia [17]. Previous studies have found that the high-altitude climate usually does not benefit the accumulation of bioactive compounds, and it will lead to plants’ low yield and unstable quality [4,18]. Furthermore, given the inherent fragility of their native alpine habitats, the overexploitation of these wild resources would pose a significant risk of ecosystem damage [19]. The utilization of these resources in ethnic medicine systems is also subject to certain limitations. For example, the scarcity and dispersion of wild resources limit their industrialization; meanwhile, the lack of quality standards and identification systems leads to inconsistent authenticity of medicinal materials in the market and insufficient stability in clinical efficacy. Therefore, given the ecological risks and limited wild resources, it is imperative to advance the artificial cultivation of *G. siphonantha* and to establish a multi-indicator quality assessment focused on iridoid glycosides. Developing a reliable quality evaluation system is essential for standardizing cultivation, promoting the sustainable use of ethnomedicinal resources, protecting fragile alpine ecosystems, and supplying high-quality raw materials for industrial applications.

In recent years, antioxidant activity has emerged as a crucial parameter for evaluating the functional values and developing potentials of traditional Chinese medicinal materials [20,21]. It is widely applied in studies related to free radical scavenging, anti-aging, and prevention or treatment of chronic diseases [22]. Among in vitro methods for assessing antioxidant activity, 1,1-diphenyl-2-picrylhydrazyl (DPPH) radical scavenging, 2,2′-azino-bis (3-ethylbenzothiazoline-6-sulfonic acid) diammonium salt (ABTS) radical scavenging, and ferric reducing antioxidant power (FRAP) assays are commonly employed due to their simplicity, sensitivity, and reproducibility [23]. Regarding antioxidant activity, iridoid glycosides in *G. siphonantha* possess well-defined structures and demonstrate strong antioxidant, anti-inflammatory, and cytoprotective activities, representing this plant’s primary pharmacological basis [24]. Previous studies have compared wild and cultivated *Gentiana* species and have indicated significant differences in the contents of active compounds and in antioxidant activity between them [8,25]. However, there is still a lack of systematic investigations into the antioxidant activity of *G. siphonantha* under different cultivation ages and harvest periods. Evaluating these aspects is essential to better understand its antioxidant properties and support rational cultivation and utilization of *G. siphonantha*.

Therefore, our study selected wild and cultivated samples from different sources. Using high-performance liquid chromatography (HPLC), we expected to quantitatively analyze the contents of five iridoid glycosides (gentiopicroside, loganic acid, sweroside, swertiamarin, and 6′-O-β-D-glucosylgentiopicroside), compare their content differences, and identify their characteristic profiles. Furthermore, we examined the accumulation characteristics of these active compounds across different cultivation ages and harvest months to identify the optimal harvest conditions. Additionally, using DPPH, ABTS, and FRAP assays, we further evaluated the antioxidant activities of the samples and the relationships between the active compounds and antioxidant activity. In this study, multivariate statistical analyses, including principal component analysis (PCA) and orthogonal partial least squares–discriminant analysis (OPLS-DA), correlation analysis, and comprehensive quality evaluation methods such as membership function and entropy weight–TOPSIS analyses, were applied for data interpretation and quality assessment. Therefore, besides a comprehensive understanding of the accumulation patterns and antioxidant capacity of *G. siphonantha* under cultivation, this study also aimed to provide a scientific basis for standardized cultivation, quality evaluation, and the sustainable utilization of *Gentiana* resources. Scientifically determined harvest periods and standardized cultivation practices not only enhance the quality of *G. siphonantha* but also alleviate the ecological pressure from wild harvesting. These measures ensure the sustainable use of ethnic medicine resources and provide a theoretical basis for the quality control and standardization of medicinal materials.

## 2. Results

### 2.1. Methodology Validation Results

**Figure 1:**
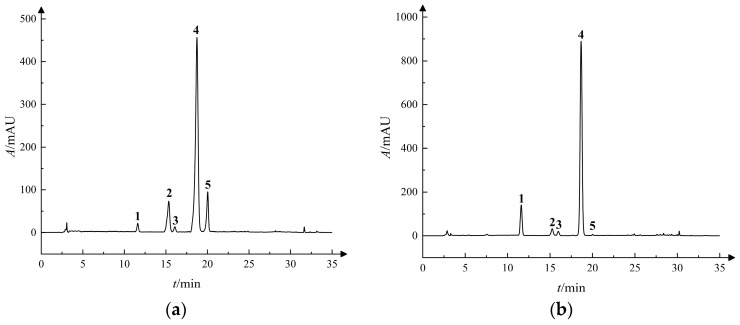
HPLC chromatographic profiles of iridoid glycosides: (**a**) standard compounds; (**b**) sample solution. Note: 1. Loganic acid; 2. Swertiamarin; 3. 6′-O-β-D-glucosylgentiopicroside; 4. Gentiopicroside; 5. Sweroside.

The dried plant samples were extracted and analyzed for iridoid glycosides using HPLC. All results are expressed as mg·g^−1^ dry weight (DW). HPLC separation results of the standards and samples are shown in Figure 1, and the linear regression equations for the five iridoid glycosides (gentiopicroside, loganic acid, sweroside, swertiamarin, and 6′-O-β-D-glucosylgentiopicroside) are presented in Table 1. All five compounds were baseline-separated with good resolution and they exhibited excellent linearity between peak area and concentration (*R*^2^ ≥ 0.9999). The limit of detection (LOD), defined at a signal-to-noise ratio (S/N) of 3:1, for each of the five target compounds was 2.00 ng, 2.25 ng, 0.50 ng, 2.25 ng, and 2.00 ng, respectively.

The results of the method validation are summarized in Table 2 and Table S1. All of the relative standard deviation (RSD) values for intra-day precision, repeatability, and stability of the five target iridoid glycosides were below 2%, and the spike recoveries ranged from 99% to 110%, demonstrating that the developed analytical method was robust, accurate, and reliable.

### 2.2. Analysis of Iridoid Glycoside Contents

#### 2.2.1. Analysis of Iridoid Glycoside Content Characteristics from Different Material Sources

**Figure 2:**
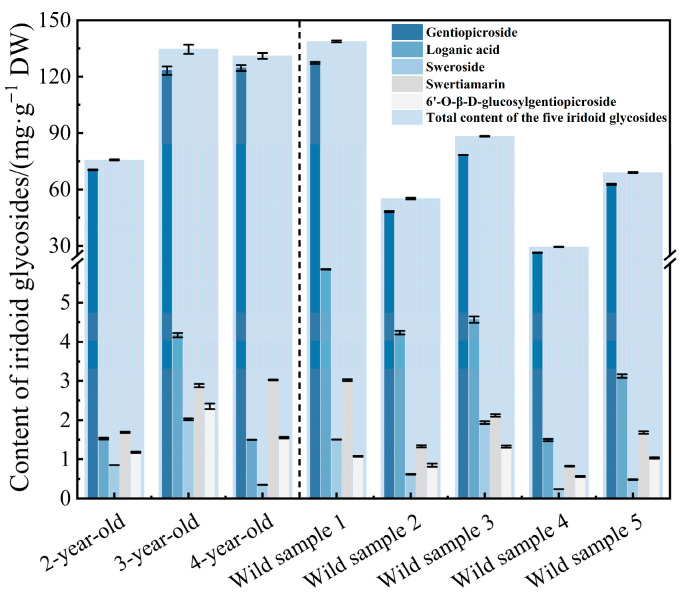
Comparison of iridoid glycoside contents between cultivated and wild samples (*n* = 3).

To elucidate the differences between cultivated and wild samples, the total contents of iridoid glycosides in cultivated *G. siphonantha* (2- to 4-year-old) and wild specimens harvested in August were comparatively analyzed. Figure 2 shows that among all cultivated samples we collected, the 3-year-old cultivated samples contained the highest total content of the five iridoid glycosides, reaching 134.60 mg·g^−1^. The cultivated *G. siphonantha* samples showed relatively consistent and generally higher iridoid glycoside contents, with lower inter-sample variability. In contrast, the contents in wild samples showed pronounced fluctuations among different collection locations. Among them, wild sample 1 exhibited the highest iridoid glycoside content (138.78 mg·g^−1^), representing an extreme value within the wild population, whereas wild sample 4 showed a much lower content of only 29.49 mg·g^−1^. Notably, wild sample 1 displayed an exceptionally high accumulation of total iridoid glycosides, representing an extreme case within the wild population rather than a general trend, which may reflect adaptive evolution to the high-altitude environment. To further verify the differences in the accumulation of iridoid compounds among different wild locations, one-way Analysis of variance (ANOVA) was performed for each individual iridoid glycoside as well as for their total content. The ANOVA results showed that the contents of all compounds in the wild samples differed extremely significantly (*p* < 0.001). Detailed ANOVA results for samples of different wild locations are available in Supplementary Table S2. Afterwards, Tukey’s HSD test was performed, and the results are presented in Supplementary Table S3. Similarly, detailed content results are shown in Table S3. This indicates that compared with wild samples, the cultivated materials, particularly those 3-year-old and 4-year-old sources, can possess relatively higher levels of iridoid glycosides and exhibit greater quality stability.

#### 2.2.2. Analysis of Iridoid Glycoside Content Characteristics of Samples Under Different Cultivation Ages

**Figure 3:**
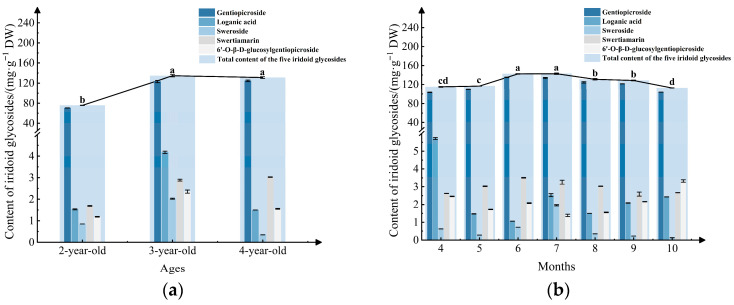
Variation in total iridoid glycoside content in *G. siphonantha* (*n* = 3): (**a**) Different cultivation ages; (**b**) Different harvest months. Note: Values in the same column followed by different lowercase letters indicate significant differences at *p* < 0.05 (Tukey’s HSD test).

To evaluate the quality differences among samples collected from different cultivation ages, we compared the total contents of iridoid glycosides in 2-year-old, 3-year-old, and 4-year-old cultivated *G. siphonantha* roots. All of these samples were harvested in August. To further verify the differences in iridoid accumulation, one-way ANOVA was performed for each individual iridoid glycoside as well as for their total content. The ANOVA results revealed extremely significant differences (*p* < 0.001) among samples from different ages for all compounds. Detailed ANOVA results for samples of different ages are available in Supplementary Table S4. Subsequently, Tukey’s HSD test was performed, and the results are presented in Supplementary Table S5. Significant differences in the total content of the five iridoid glycosides are shown in Figure 3a. Based on the above statistical results, the total content of iridoid glycosides showed a non-monotonic trend (first increasing and then slightly decreasing) over the cultivation duration (Figure 3a). Detailed quantitative values of individual compounds and the total content are provided in Supplementary Table S5. Among them, the 3-year-old samples contained the highest level (134.60 mg·g^−1^), exceeding those of the 2-year-old and 4-year-old samples, suggesting that the third cultivation age (i.e., the 3-year-old stage) represents the major accumulation age of iridoid glycosides.

These findings show that among the cultivated samples, the 3-year-old samples demonstrated the highest quality and greatest application potential. Therefore, *G. siphonantha* can accumulate bioactive constituents under standardized cultivation practices.

#### 2.2.3. Analysis of Iridoid Glycoside Content Characteristics of Samples Harvested in Different Months

To further analyze the accumulation pattern of iridoid glycosides in cultivated *G. siphonantha* harvested across different months, we systematically examined the total contents of iridoid glycosides in 4-year-old samples that were monthly collected from April to October. To examine the differences in iridoid accumulation, one-way ANOVA was conducted for each individual iridoid glycoside as well as for their total content. The results revealed extremely significant differences (*p* < 0.001) among samples from different months for almost all compounds. The only exception was swertiamarin, which still showed a highly significant difference (*p* < 0.01). Detailed ANOVA results for samples collected in different months are provided in Supplementary Table S6. Following this, Tukey’s HSD test was carried out, and the corresponding results are presented in Supplementary Table S7. Significant differences in the total content of the five iridoid glycosides are shown in Figure 3b. As presented in Figure 3b, the iridoid glycoside content revealed an inverted “V”-shaped trend, in which the total content increased initially and then decreased over time. Specifically, the total iridoid glycoside content was 115.01 mg·g^−1^ in April, which slightly increased in May, rose sharply in June (reaching 142.44 mg·g^−1^), and reached a maximum value of 142.72 mg·g^−1^ in July. After this increase, the content gradually declined to 112.62 mg·g^−1^ in October. Detailed quantitative values of individual compounds and the total content are provided in Supplementary Table S7. This pattern reveals a dynamic model of secondary metabolite accumulation, which is characterized by continuous biosynthesis during the growth and maturation stages with a peak at maturity [26]. Regarding this medicinal material accumulation of iridoid glycosides, June–July represents the peak accumulation period of iridoid glycosides in 4-year-old cultivated samples. Therefore, June–July can be preliminarily considered the optimal harvest period for cultivated *G. siphonantha*.

### 2.3. Quality Evaluation

#### 2.3.1. PCA

**Figure 4:**
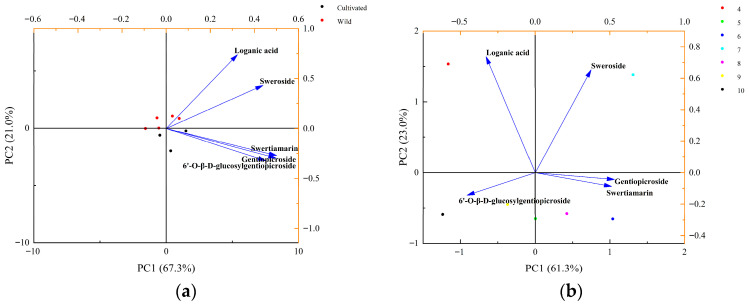
PCA distribution map and factor load map: (**a**) Cultivated and wild samples; (**b**) Cultivated samples from different months.

To evaluate the variations in the five iridoid glycosides of *G. siphonantha*, our study conducted PCA. Holistically, a significant disparity was observed between cultivated and wild samples (Figure 4a), and differences were also evident among cultivated samples collected in different months (Figure 4b).

Principal components were extracted according to the criterion of eigenvalues greater than 1. Figure 4a shows that both of the first two principal components (PC1 is 67.3% and PC2 is 21.0%) collectively explained 88.3% of the total variance. This suggests that this PCA model possessed strong representativeness and reliability. The score plot revealed that cultivated samples were primarily distributed along the positive axis of PC1, whereas wild samples were on the negative axis (Figure 4a), suggesting that cultivated samples were effectively distinguished from wild ones in terms of PC1. Moreover, the orientation of the cultivated samples was consistent with the loading directions of the iridoid glycosides (Figure 4a), implying that the overall levels of these compounds were higher in cultivated materials than those in their wild counterparts. This demonstrates that the cultivation of *G. siphonantha* is feasible.

As illustrated in Figure 4b, the cumulative contribution of the first two principal components (PC1 is 61.3% and PC2 is 23.0%) reached 84.3%, further confirming the model’s reliability shown in Figure 4b. Cultivated samples collected in different months showed a moderate degree of differences in the PCA space. This suggests that *G. siphonantha*’s compositional contents change as the plant grows. At the early growth stage (April), samples were distributed in the second quadrant along the negative axis of PC1 and were strongly associated with loganic acid, indicating a relatively high content of this compound. During the mid-growth stage (May–August), samples were mainly located in the positive direction of PC1, corresponding to the progressive accumulation of gentiopicroside, sweroside, and swertiamarin. In the late growth stage (September–October), samples shifted toward the third quadrant along the negative axis of PC1, characterized by a higher relative abundance of 6′-O-β-D-glucopyranosyl gentiopicroside and a noticeable decline in the total iridoid glycoside content compared with the mid-growth stage. The above analytical results are generally consistent with the findings from the content analysis.

#### 2.3.2. OPLS-DA

**Figure 5:**
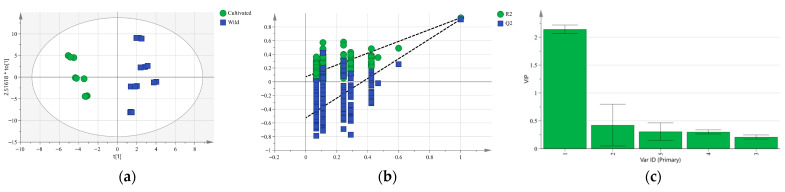
OPLS-DA analysis of *G. siphonantha* from different sources: (**a**) Scores plot; (**b**) Cross-validation results; (**c**) VIP plot. Note: In plot (**b**), dashed lines show the Y-intercept thresholds of *R*^2^ and *Q*^2^ from the permutation test, used to assess model overfitting. In plot (**c**), compounds 1–5 represent gentiopicroside, loganic acid, sweroside, swertiamarin, and 6′-O-β-D-glucopyranosyl gentiopicroside, respectively.

**Figure 6:**
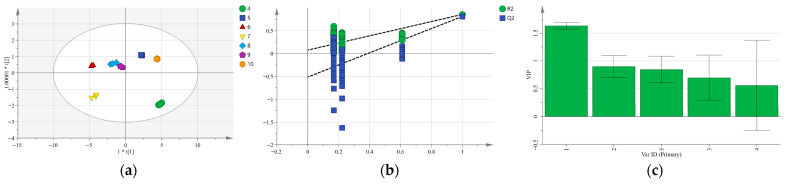
OPLS-DA analysis of *G. siphonantha* from different months: (**a**) Scores plot; (**b**) Cross-validation results; (**c**) VIP plot. Note: In plot (**b**), dashed lines show the Y-intercept thresholds of *R*^2^ and *Q*^2^ from the permutation test, used to assess model overfitting. In plot (**c**), compounds 1–5 represent gentiopicroside, loganic acid, sweroside, swertiamarin, and 6′-O-β-D-glucopyranosyl gentiopicroside, respectively.

To further investigate the intra-group variations among *G. siphonantha* samples from different sources and growth months, we conducted a supervised OPLS-DA (see Figure 5 and Figure 6).

As shown in Figure 5a, samples from cultivated and wild sources were clearly distinguished along the horizontal axis of the score plot. The model exhibited excellent performance, with the independent variable fitting index (*R*^2^*X*) of 1.000, dependent variable fitting index (*R*^2^*Y*) of 0.933, and predictive ability (*Q*^2^) of 0.912, all exceeding the 0.5 threshold, thus confirming the model’s strong explanatory power and predictive reliability. The 200-permutation test (Figure 5b) yielded a negative intercept for the *Q*^2^ regression line on the vertical axis, indicating the absence of overfitting and validating the model’s robustness. Regarding variable importance in projection (VIP) analysis, previous studies have found that variables with higher VIP values contribute more substantially to sample classification [27]. As illustrated in Figure 5c, gentiopicroside showed the highest VIP scores, suggesting that this compound can be considered the primary contributor or factor to quality differentiation of samples from different sources. Consistently, the OPLS-DA score plot in Figure 6 also demonstrated a clear separation among samples harvested in different months. The OPLS-DA results demonstrate that the supervised discriminant model effectively separates cultivated from wild samples, as well as samples harvested in different months, with no evidence of overfitting and satisfactory model stability. Moreover, the model outcomes further elucidate the variation trajectories of chemical constituents and associated quality attributes in cultivated *G. siphonantha*.

The relatively large standard deviations observed in the VIP plots (Figure 5c and Figure 6c) do not indicate analytical instability but rather reflect pronounced biological variability among the samples and variability in variable importance across different sample groups. For the comparison between wild and cultivated samples (Figure 5c), the higher variability is mainly attributable to the intrinsic heterogeneity of wild populations. Wild *G. siphonantha* plants are exposed to highly variable environmental conditions, including differences in altitude, soil properties, microclimate, and ecological stress, which strongly influence the biosynthesis and accumulation of iridoid glycosides [28]. Consequently, the contribution of individual compounds to group discrimination varies markedly among wild samples, resulting in larger standard deviations in VIP values. In the case of samples harvested in different months (Figure 6c), the observed variability is likely related to seasonal and developmental regulation. During the growth period from April to October, the biosynthesis of iridoid glycosides undergoes dynamic changes, and the relative importance of individual compounds for sample discrimination differs across growth stages. This temporal heterogeneity is therefore reflected as increased variability in VIP scores.

#### 2.3.3. Membership Function Analysis

Using membership function method, we conducted a quality evaluation of iridoid glycosides in *G. siphonantha* samples collected from different sources and months (see Table 3 and Table 4). Table 3 shows membership function values of the cultivated and wild samples, which ranged from 0.000 to 0.902. This wide spectrum of membership function values suggests significant differences in quality among the various samples. Notably, the 3-year-old cultivated samples displayed higher membership function values across all components, particularly for gentiopicroside, sweroside, swertiamarin, and 6′-O-β-D-glucosylgentiopicroside, with individual values approaching or reaching 1.000 and an overall membership function value of 0.902. This indicates that the 3-year-old cultivated plants contain the largest accumulation of active compounds and therefore they have superior quality. In contrast, the 2-year-old cultivated samples, wild sample 2, and wild sample 5 showed lower membership function values, corresponding to their lower compound contents and moderate quality. All of the membership function values of wild sample 4 were zero, indicating its poor quality. Moreover, wild samples exhibited significant differences in their membership function values, reflecting low consistency in quality. Certain samples, such as the 4-year-old cultivated samples, wild sample 1, and wild sample 3, exhibited intermediate levels, suggesting moderate accumulation of active compounds. Collectively, these results suggest that 3-year-old cultivated materials contain the highest quality; therefore they can be recommended as the optimal cultivation age.

#### 2.3.4. Entropy Weight–TOPSIS Analysis

Using the entropy weight–TOPSIS method, a comprehensive evaluation was conducted for samples from different sources. The contents of five iridoid glycosides were used as variables, and the data were normalized with the greater the content, the better the type. The information entropy (*E_j_*), information utility value (*D_j_*), and indicator weights (*W_j_*) were then calculated. The *W_j_* calculation results were shown in Table 5. The results showed that the *W_j_* of loganic acid is the highest (*W_j_* = 0.290), followed by sweroside, indicating that these indicators contribute the most to the comprehensive evaluation. The weighted decision matrix was subsequently obtained by multiplying the normalized data by the indicator weights. The positive and negative ideal solutions were then determined. After that, the Euclidean distances of each sample to the ideal solutions (*D_i_*^+^, *D_i_*^−^) were calculated, and the closeness coefficient (*C_i_*) was derived for comprehensive ranking, as presented in Table 6. The results showed that the 3-year-old samples had the highest *C_i_* value (0.782), markedly outperforming the other age groups, suggesting that 3-year cultivation stage is the optimal harvest age.

Similarly, according to the entropy weight–TOPSIS method, the samples in different months are comprehensively evaluated, and the *W_j_* calculation results are shown in Table 7. Sweroside received the highest *W_j_* (0.313), followed by loganic acid and swertiamarin, indicating that these indicators have greater explanatory power in the overall evaluation model. According to the *C_i_* values (Table 8), the July samples exhibited the highest *C_i_* value (0.605), followed by June (*C_i_* = 0.488), suggesting that samples collected in June–July possessed the best overall quality, which is consistent with the ranking based on iridoid glycosides content. Through the entropy weight–TOPSIS comprehensive evaluation, the relative advantages and disadvantages of the samples in each month can be objectively reflected, which provides a reliable basis for determining the best harvest period. Overall, June–July can be considered the optimal harvest period for *G. siphonantha*. This finding is consistent with the results of the membership function analysis, based on which the 3-year cultivation stage is identified as the optimal harvest age, and June–July of the fourth cultivation year was found to be the most suitable harvest period.

### 2.4. Antioxidant Activity Evaluation

The antioxidant activities of *G. siphonantha* samples across different cultivation ages and harvest months were comprehensively evaluated by employing three complementary antioxidant assays: DPPH, ABTS, and FRAP.

#### 2.4.1. Evaluation of Antioxidant Activity Under Different Cultivation Ages

**Figure 7:**
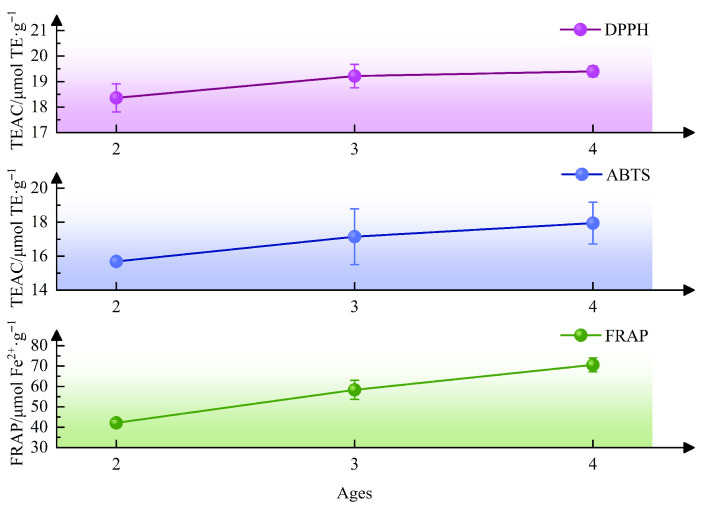
Antioxidant activities of *G. siphonantha* harvested at different cultivation ages (*n* = 3).

The antioxidant activities of *G. siphonantha* samples harvested in August across different cultivation ages were evaluated. As shown in Figure 7, the trolox equivalent antioxidant capacity (TEAC) values analyzed through the DPPH assay increased from 15.30 μmol TE·g^−1^ in 2-year-old samples to 20.69 μmol TE·g^−1^ in 4-year-old samples. The ABTS assay results exhibited a similar upward trend, with TEAC values rising from 12.44 μmol TE·g^−1^ in 2-year-old samples to 17.03 μmol TE·g^−1^ in 4-year-old samples. FRAP assay results indicated a progressive increase in ferric-reducing antioxidant power with cultivation age, from 25.98 μmol Fe^2+^·g^−1^ in 2-year-old samples to 50.88 μmol Fe^2+^·g^−1^ in 4-year-old samples. These consistent trends across all three assays demonstrate that antioxidant capacity in *G. siphonantha* progressively enhances with increasing cultivation ages, reflecting the gradual accumulation of bioactive antioxidant compounds during plant growth and maturation.

#### 2.4.2. Evaluation of Antioxidant Activity Under Different Harvest Months

**Figure 8:**
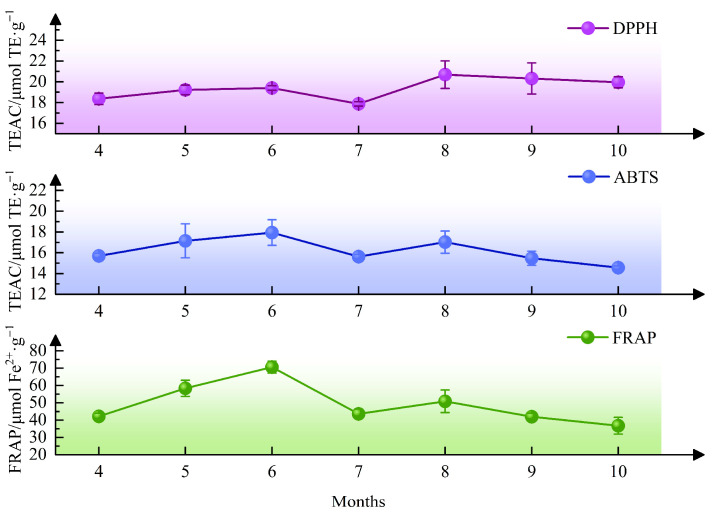
Antioxidant activities of *G. siphonantha* harvested at different harvest months (*n* = 3).

The antioxidant activities of the medicinal parts of 4-year-old *G. siphonantha* exhibited noticeable fluctuations throughout the harvest months (Figure 8). In the early growth stage (from April to June), antioxidant activity steadily increased. In July, there was a slight decline. Subsequently, TEAC and FRAP values rapidly rose in August, reaching a second peak, and finally stabilized from September to October. Overall, the antioxidant capacity displayed a bimodal fluctuation pattern throughout the growth period, with maxima observed in June and August.

### 2.5. Correlation Analysis Between Iridoid Glycosides and Antioxidant Activity

#### 2.5.1. Gray Relational Analysis

The data were normalized using the mean-scaling method to render them dimensionless, followed by gray relational analysis (GRA) to evaluate the relationships between the contents of individual iridoid glycosides, total iridoid glycoside content, and antioxidant activities. In this analysis, the DPPH, ABTS, and FRAP antioxidant activities were set as the reference sequences, whereas the contents of the five individual iridoid glycosides and their total content were designated as the characteristic sequences. The correlation degree between each characteristic sequence and the reference sequences was calculated and ranked. As shown in Table 9, the gray relational coefficients (*γ*) between each iridoid glycoside and the DPPH, ABTS, and FRAP antioxidant activities were all greater than 0.540, indicating strong associations between these compounds and the measured antioxidant activities.

#### 2.5.2. Spearman’s Rank Correlation Analysis

**Figure 9:**
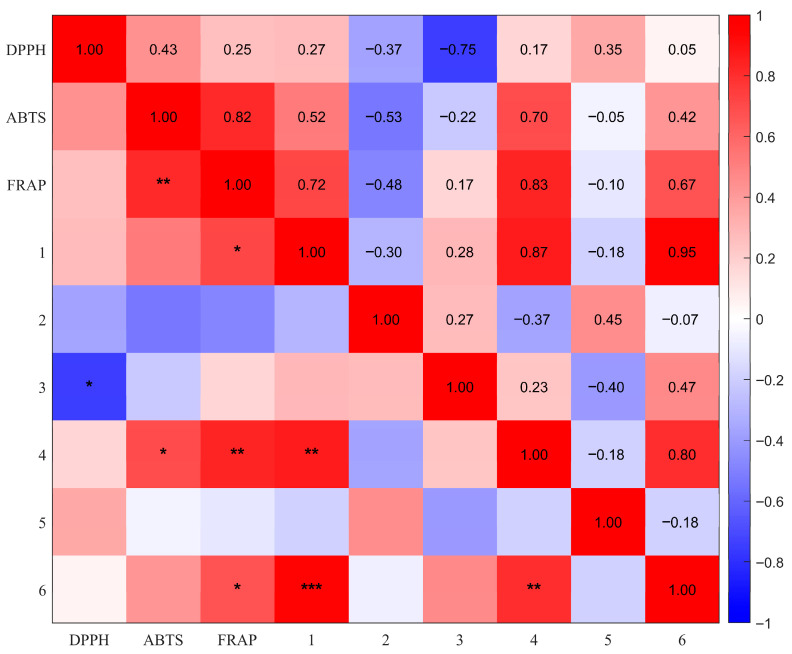
Correlation analysis between antioxidant indicators and the contents of five iridoid glycosides and their total amount. Note: Compounds 1–6 represent gentiopicroside, loganic acid, sweroside, swertiamarin, 6′-O-β-D-glucopyranosyl gentiopicroside and content of iridoid glycosides, respectively. * *p* < 0.05; ** *p* < 0.01; *** *p* < 0.001.

To investigate the relationships between iridoid glycosides and in vitro antioxidant activities of *G. siphonantha*, Spearman’s rank correlation analysis was performed, and the results are shown in Figure 9. Gentiopicroside exhibited a significant positive correlation with FRAP (*ρ* = 0.72, *p* < 0.05), while swertiamarin showed significant positive correlations with ABTS (*ρ* = 0.70, *p* < 0.05) and FRAP (*ρ* = 0.83, *p* < 0.01), respectively. The total content of iridoid glycosides was significantly positively correlated with FRAP (*ρ* = 0.67, *p* < 0.05). Furthermore, the total iridoid glycoside content was positively correlated with all three antioxidant indicators, suggesting that iridoid glycosides dominated by swertiamarin and related compounds may play a key role in the antioxidant activity of *G. siphonantha*, potentially serving as the primary chemical basis underlying its bioactivity.

Table 4 shows the membership function values of five iridoid glycosides in 4-year-old *G. siphonantha* samples collected across different months. Their overall membership function values ranged from 0.204 to 0.600, reflecting big fluctuations and indicating significant differences in material quality among different harvest months. In terms of the overall membership function values, samples harvested in July showed the highest score (0.600), followed by those collected in June (0.537) and April (0.374), while samples harvested from other months (including May, August, September, and October) showed comparatively lower values. The July samples were distinctively characterized by high levels of gentiopicroside, sweroside, and swertiamarin. This distinction reveals an accumulation pattern of iridoid glycosides, which was consistent with the above-mentioned PCA results. Both gentiopicroside and swertiamarin reached their maximum concentrations in July, suggesting that this stage represents the peak period of active compound biosynthesis. At the same time, the membership function value in June was second only to that in July, and the contents of gentiopicroside and swertiamarin were the highest in that month, which was consistent with the PCA results. In contrast, samples harvested in May, August, September, and October exhibited relatively lower comprehensive quality and reduced metabolite accumulation. Collectively, these findings indicate that June–July represents the optimal harvest period for 4-year-old cultivated *G. siphonantha* materials in terms of iridoid glycoside enrichment and overall quality performance.

## 3. Discussion

As an important ethnomedicinal resource, the quality stability of *G. siphonantha* is directly linked to the consistency of its medicinal efficacy and its resources’ sustainable utilization. The stability and controllability of its bioactive constituents are essential for ensuring reproducible efficacy as well as meeting the requirements of modern quality standardization [29]. To evaluate cultivated materials’ quality stability, this study first conducted a comparative analysis of iridoid glycoside contents between cultivated and wild samples. The superior quality stability observed in cultivated *G. siphonantha* refers primarily to their lower inter-sample variability and more consistent iridoid glycoside levels. In contrast, wild samples exhibited markedly greater heterogeneity in iridoid glycoside accumulation. These wild samples’ differences are likely due to variations in growth age, complex environmental conditions, and genetic diversity [30]. In particular, wild sample 1 showed an exceptionally high iridoid glycoside content, representing an extreme value within the wild population. Although detailed environmental parameters were not available in the present study, previous studies have demonstrated that environmental factors such as altitude, temperature, soil conditions, and ecological stress can strongly influence iridoid biosynthetic pathways in medicinal plants [31,32]. The pronounced accumulation observed in wild sample 1 may therefore be associated with a specific combination of favorable or stress-related environmental conditions. Importantly, such extreme cases are highly informative and may provide valuable guidance for future cultivation strategies. Identifying and simulating key environmental drivers associated with high iridoid glycoside accumulation could contribute to the optimization of *G. siphonantha* cultivation conditions aimed at maximizing iridoid biosynthesis.

In this study, one-way ANOVA was performed for each individual iridoid glycoside as well as for their total content, followed by Tukey’s HSD test for multiple pairwise comparisons. The results demonstrated that almost all compounds differed extremely significantly among samples from different cultivation years, collection months, and wild locations, confirming the reliability and robustness of our findings. Our further comparative analysis of samples harvested in the same month but with different cultivation ages reveals that the total iridoid glycoside content was highest in the 3-year-old cultivated samples. In contrast, the 2-year-old samples showed relatively low levels, as the plants were still in the early vegetative growth stage during which metabolic resources are primarily allocated to structural development rather than secondary metabolism. In the fourth year of cultivation, the total content of iridoid glycosides slightly declined, which may be attributed to reduced metabolic activity associated with physiological senescence, coupled with changes in substance transport and allocation [33]. Consequently, the levels of active compounds in 4-year-old samples were lower than those in 3-year-old samples, a similar phenomenon observed in studies on cultivated *G. crassicaulis* [34]. Among the cultivated samples, the 3-year-old plants exhibited the highest total content of iridoid glycosides, and ANOVA revealed a significant difference compared with the 2-year-old cultivated samples. Therefore, the 3-year cultivation stage can be preliminarily considered the optimal harvest age. Although the 3-year-old samples exhibited the highest quality, the 4-year-old cultivated materials were still selected for our further analysis of harvest-month effects due to their greater representativeness in practical cultivation [35,36]. Generally, regarding harvest-month effects analysis, within a single growth cycle of cultivated *G. siphonantha*, plants remain in the early growth stage during April-May, when both biosynthesis and accumulation of iridoid glycosides are limited. During the peak biosynthesis stage (June–July), the contents of iridoid glycosides reach their maximum, likely associated with concentrated rainfall, enhanced photosynthetic activity, accelerated nutrient transport to roots, and increased flux through secondary metabolic pathways [37]. More specifically, this temporal trend is likely associated with the physiological status of the plant, as early to mid-summer represents a phase of vigorous growth and active secondary metabolite biosynthesis. Environmental conditions such as increased sunlight, higher temperatures, and enhanced photosynthetic activity during this period may further promote the iridoid pathway, thereby contributing to the elevated accumulation of iridoid glycosides. From August to September, as temperatures gradually decrease, leaf senescence progresses and root development largely ceases [38]. By October, aging and environmental constraints such as low temperature further reduce the plant’s metabolic activity, leading to a decline in secondary metabolite concentrations [39]. This accumulation pattern aligns with the seasonal changes in iridoid glycosides, which were also observed in other *Gentiana* species. This pattern suggests that their biosynthesis is collectively impacted by both developmental stages and environmental factors across different growing months [25,40]. In addition, similar seasonal fluctuations have been observed in other *Gentiana* species [25,40], indicating that this phenomenon is biologically consistent and species-related. The ANOVA results corroborated these observations by showing statistically significant differences among months, with June–July samples exhibiting markedly higher contents than those from the remaining periods. This statistical evidence, together with the content distribution, reinforces the conclusion that June–July is the optimal harvest season for achieving maximum iridoid accumulation.

This study employed three approaches to evaluate *G. siphonantha* sample quality: PCA, OPLS-DA, membership function analysis, and entropy weight–TOPSIS analysis. PCA, an unsupervised pattern recognition method, can reveal the overall variation trends and underlying distribution patterns among samples [41]. OPLS-DA, a supervised classification approach, emphasizes group differences and is suitable for further analyzing the relationships between variables and sample categories [42]. The membership function analysis enables quantitative normalization across multiple indicators and makes it easier to conduct a comprehensive ranking [43]. The entropy weight–TOPSIS analysis combines objective indicator weighting with comprehensive sample ranking based on their closeness to ideal solutions [44]. The results obtained from these four approaches were generally consistent. Firstly, we find that the 3-year cultivation stage represents the optimal harvest age. Harvesting can be carried out after three years of cultivation. Secondly, our results show that the 4-year-old samples harvested in June–July exhibited the highest iridoid glycoside content, suggesting that June–July is the most suitable harvest period. Specifically, samples harvested in July showed a higher content of the target compounds, whereas those collected in June offered a better balance between content and antioxidant activity. These findings are consistent with the traditional harvesting periods of *Gentiana* species [38,45].

In addition, our study analyzed the antioxidant activities of *G. siphonantha*. Although the medicinal parts of *G. siphonantha*, namely the roots, grow in dark environments, they are still exposed to various external stresses such as soil pathogens, heavy metal ions, and oxidative stress. To maintain cellular homeostasis and resist the adverse conditions, plants activate root antioxidant systems and synthesize secondary metabolites with radical-scavenging capacities, thereby enhancing their antioxidant defense [46]. Our study demonstrated consistent results across three antioxidant assays (including DPPH, ABTS and FRAP), indicating that the antioxidant capacity of cultivated samples of *G. siphonantha* progressively increases with their cultivation ages. This progressive increase suggests a gradual accumulation of antioxidant compounds in the roots. We find that the antioxidant activity of the 4-year-old samples shows certain fluctuations throughout different harvest months. Specifically, from April to June, the plants’ metabolic pathways related to secondary metabolism become increasingly active, resulting in a steady enhancement of antioxidant capacity. Although the total iridoid glycoside content reached its maximum in July, the antioxidant activities evaluated by DPPH, ABTS, and FRAP assays did not show a synchronous increase. This phenomenon may be attributed to the multifactorial nature of antioxidant activity at the extract level and the non-linear relationship between individual metabolite classes and antioxidant indices [47,48]. In addition, July corresponds to a period of vigorous vegetative growth, during which the accumulation of iridoid glycosides may be prioritized, whereas the accumulation of other antioxidant-related metabolites may not be synchronized, leading to a temporary decoupling between iridoid glycoside content and antioxidant activity [47,49]. In August, the antioxidant activity increased again to reach another peak, suggesting that plants may activate compensatory metabolic mechanisms in response to environmental stress, therefore enhancing antioxidant system activity and promoting the bioactive substances’ synthesis and accumulation [50]. In the following growth stage after August, underground metabolism tended to stabilize, leading to a new balance between the antioxidant compounds’ accumulation and consumption. Accordingly, compared with other months, samples harvested in both June and August showed stronger antioxidant activities. This further confirms that June–July can be considered the optimal harvest period, with both the total content of iridoid glycosides and antioxidant activities being relatively high in June. Previous studies have reported that methanolic extracts of *Gentiana* roots exhibit notable antioxidant activity. For instance, ethanol extracts of *G. lutea* roots have been reported to exhibit significant DPPH and ABTS radical scavenging activities, with a DPPH TEAC value of 15.89 μmol TE·g^−1^ DW. Similarly, methanolic root extracts of *G. kurroo* have been shown to possess strong DPPH and FRAP scavenging capacities. In this context, the antioxidant activity observed for *G. siphonantha* roots in the present study is generally comparable to that reported for other *Gentiana* species, supporting the relevance of our results within the genus. Importantly, unlike previous studies that mainly focused on single-time-point samples, the present study systematically evaluated samples collected across multiple months and years, revealing clear temporal patterns in antioxidant activity.

*Gentiana* species are characterized by a high abundance of iridoid glycosides, which are considered their major and characteristic secondary metabolites. Increasing evidence suggests that iridoid compounds possess intrinsic antioxidant potential. For example, iridoid glycosides isolated from *Gentiana* rhizomes have been shown to effectively scavenge DPPH radicals [51], and gentirigeoside B has been reported to exert antioxidant effects through the modulation of oxidative stress–related signaling pathways [52]. Based on these studies, the relationship between the iridoid glycosides and each antioxidant activity was analyzed using GRA and Spearman’s rank correlation. The GRA results revealed high correlation coefficients (*γ* > 0.540) between the contents of each iridoid glycoside and antioxidant indicators (DPPH, ABTS, and FRAP). Among them, ABTS showed the strongest correlation with swertiamarin (*γ* = 0.914), while FRAP displayed strong associations with swertiamarin, gentiopicroside, and the total iridoid glycoside content. In addition, Spearman’s rank correlation analysis further confirmed these results, indicating that iridoid glycosides such as swertiamarin may play key roles in the antioxidant activity of *G. siphonantha*. This finding is consistent with previous literature [9,10] reporting that swertiamarin exhibits antioxidant activity. Although the antioxidant activity of methanolic extracts reflects the combined contribution of multiple constituents, the significant correlations observed in the present study between iridoid glycoside profiles and antioxidant indices suggest that iridoid glycosides may contribute to antioxidant performance at the extract level, possibly through indirect or synergistic effects [9,10,11,12,13,14]. Taken together, both GRA and Spearman’s rank correlation analysis have demonstrated strong correlations between iridoid glycoside contents and antioxidant activities. These findings are consistent with those in previous studies [53,54,55], which reported that iridoid glycosides represent the bioactive substances responsible for the antioxidant potential of *Gentiana* species.

*G. macrophylla* is an officially recorded medicinal species in the ChP [15]. Previous studies have suggested that *G. siphonantha* may serve as a potential alternative resource to *G. macrophylla* based on their close taxonomic relationship and chemical similarity [6,7]. The present study provides additional chemical evidence supporting this perspective, particularly through systematic analysis of iridoid glycoside profiles. The results indicate that, at the chemical level, *G. siphonantha* possesses a composition and accumulation pattern of iridoid glycosides comparable to those reported for *G. macrophylla*. Nevertheless, it should be emphasized that formal substitution for a pharmacopoeial species would require comprehensive pharmacological, toxicological, and clinical validation. Therefore, while *G. siphonantha* demonstrates promising chemical potential as an alternative resource, further studies are necessary before any official substitution can be recommended [9,10,11,12,13,14]. It should be noted that this study has certain limitations. First, phenolic compounds and flavonoids, which are widely recognized as major contributors to antioxidant activity, were not systematically quantified; therefore, their potential contributions to the observed antioxidant performance cannot be excluded. Nevertheless, the observed correlations, together with previous studies reporting the contribution of iridoid glycosides to antioxidant activity, suggest that iridoid glycosides represent important contributors and potential key material bases underlying the antioxidant activity of *G. siphonantha* roots. Second, the antioxidant evaluation was based solely on in vitro chemical assays and conducted exclusively on cultivated *G. siphonantha* samples. Further verification using in vivo or cell-based models, as well as comparative assessments including wild samples, is required to better reflect the pharmacological relevance of these findings.

## 4. Materials and Methods

### 4.1. Instruments

The following instruments and equipment were used in this study: a high-performance liquid chromatograph (Infinity 1260, Agilent Technologies, Santa Clara, CA, USA), a centrifuge (5810R, Eppendorf, Hamburg, Germany), a microplate reader (Epoch2, BioTek, Winooski, VT, USA), an analytical balance (ME104, 0.0001 g, Mettler Toledo, Greifensee, Switzerland), an ultrapure water system (Molecular, Shanghai, China), an Eclipse Plus C18 column (4.6 × 250 mm, 5 μm, i.d., Agilent Technologies, USA), a grinder (Tianjin Test Instrument Co., Ltd., Tianjin, China), a CNC ultrasonic cleaner (KH-500DE, Kunshan Hechuang Ultrasonic Instrument Co., Ltd., Kunshan, China), an oven (DHG-9245A, Shanghai Yiheng Technology Co., Ltd., Shanghai, China), and a desiccator.

### 4.2. Chemicals and Materials

Our study employed the following chemicals and materials: Methanol (HPLC grade, Supelco^®^, Bellefonte, PA, USA), methanol (analytical grade, Hubei Futon Science & Technology Co., Ltd., Wuhan, China), acetonitrile (HPLC grade, SAFR, Handan, China), acetic acid (analytical grade, Fuyu Chemical, Shanghai, China), gentiopicroside reference standard (Chengdu Alpha Biotech Co., Ltd., Chengdu, China, batch number: AB1317-0020, purity ≥ 98%), loganic acid reference standard (National Institute for Food and Drug Control, Beijing, China, batch number: 111,865–202,406, purity ≥ 98%), sweroside reference standard (prepared in-house, purity ≥ 98%), swertiamarin reference standard (National Institute for Food and Drug Control, China, batch number: 110,785–202,205, purity ≥ 98%), 6′-O-β-D-glucosylgentiopicroside reference standard (Chengdu Alpha Biotech Co., Ltd., batch number: AB1241-0020, purity ≥ 98%), DPPH radical scavenging assay kit (cat. no.: A153-1-1, Nanjing Jiancheng Bioengineering Institute, Nanjing, China), total antioxidant capacity (T-AOC) ABTS assay kit (cat. no.: A015-2-1, Nanjing Jiancheng Bioengineering Institute, Nanjing, China), and total antioxidant capacity (T-AOC) FRAP assay kit (cat. no.: A015-3-1, Nanjing Jiancheng Bioengineering Institute, Nanjing, China).

Experimental *Gentiana siphonantha* Maxim. ex Kusn. samples were collected from both our research project’s cultivation base and wild habitats, with detailed information provided in Table 10. All cultivated samples were collected from the same cultivation stand of approximately 6667 m^2^ (≈0.67 ha) located in Huangzhong District, Xining City, Qinghai Province, where *G. siphonantha* has been continuously cultivated for multiple consecutive years. To ensure comparability across years and months, environmental conditions and field management practices remained consistent. Based on previous studies on *Gentiana* species and related medicinal plants [7,38,45,56,57], the optimal harvest period was determined by month, which is a common and meaningful approach for evaluating phytochemical composition. Accordingly, all cultivated samples were collected at fixed times each month, with 20–30 individual plants harvested per sampling. The collected plants were brought back to the laboratory, cleaned to remove soil and impurities, air-dried in the shade, oven-dried at 45 °C to constant weight, pulverized, passed through a 50-mesh sieve, and mixed to form composite samples, which were finally stored in a desiccator at room temperature in the dark until analysis. Because the exact age of wild plants could not be determined, approximately 30 individuals were sampled at each location to ensure representativeness. Wild samples were similarly cleaned, air-dried in the shade, oven-dried at 45 °C to constant weight, pulverized, passed through a 50-mesh sieve, and mixed to form composite samples, which were stored under the same conditions. This approach minimizes the potential influence of age variation among wild plants and ensures the uniformity, representativeness, and suitability of all samples for subsequent analyses. The original plant materials were taxonomically authenticated as *Gentiana siphonantha* Maxim. ex Kusn. by Professor Yuanwen Duan, Kunming Institute of Botany, Chinese Academy of Sciences.

The cultivation location (latitude 36.4434° N, longitude 101.5136° E; altitude 2901.23 m) is located in Huangzhong District, Xining, Qinghai Province. It belongs to a continental plateau climate, with a mean annual temperature of 4.6 °C and annual precipitation of 500–650 mm. The soil is mainly chernozem soil with a pH of 8.20 and organic matter content of 6.04 g·kg^−1^. Other physicochemical properties, including total N, P, and K, alkali-hydrolyzable N, available P and K, total salt content, and the carbon-to-nitrogen (C/N) ratio, were determined according to the NY/T 1121 series of methods [58]. Heavy-metal contents (Pb, Cd, Hg, and As) were measured by ICP-MS following HJ 804-2016 [59], and all were below the safety limits specified in the Chinese Soil Environmental Quality Standard (GB 15618-2018 [60]). Detailed measurement results are provided in Supplementary Table S8. For the wild samples, the collection locations were located in Qinghai Province, which are typical alpine environments consisting of meadows, shrub-steppe regions, and areas with hillsides, riverbanks, grasslands, and shrubs and are characterized by cool temperatures, low precipitation, and minimal anthropogenic contamination. All sampling locations in this study are suitable for the cultivation of the medicinal plant.

### 4.3. Solution Preparation

#### 4.3.1. Preparation of Sample Solutions

An accurately weighed 0.5000 ± 0.0001 g of powdered *G. siphonantha* sample was placed into a stoppered conical flask before 20 mL of methanol was precisely added. After the mixture was subjected to ultrasonic extraction for 30 min, allowed to cool down to room temperature, it was then transferred to a centrifuge tube. Centrifugation was performed at 4000 rpm for 20 min, and then the resulting supernatant was transferred to a volumetric flask and brought to a final volume of 20 mL with methanol. The prepared solution was used for subsequent HPLC analysis. All results are expressed as mg·g^−1^ DW.

#### 4.3.2. Preparation of Mixed Reference Solutions

The five iridoid glycoside reference standards (gentiopicroside, loganic acid, sweroside, swertiamarin, and 6′-O-β-D-glucosylgentiopicroside) were accurately weighed and dissolved in methanol to prepare individual stock solutions at appropriate concentrations. These solutions were subsequently combined and diluted to obtain a mixed standard solution with final concentrations of 7.5 mg·mL^−1^, 0.3 mg·mL^−1^, 0.2 mg·mL^−1^, 0.3 mg·mL^−1^, and 0.2 mg·mL^−1^, respectively. The mixed standard solution was stored at 4 °C under light-protected conditions until analysis.

### 4.4. Determination of Iridoid Glycosides

The contents of gentiopicroside, loganic acid, sweroside, swertiamarin, and 6′-O-β-D-glucosylgentiopicroside in the samples were determined by HPLC. The filtrate was injected into the HPLC system and analyzed under the following chromatographic conditions. Chromatographic separation was performed on an Eclipse Plus C18 column (4.6 × 250 mm, i.d. 5 μm, Birmingham, AL, USA). The mobile phase consisted of solvent A (0.1% aqueous acetic acid) and solvent B (acetonitrile) with a flow rate of 1.0 mL·min^−1^. The column temperature was maintained at 30 °C, the injection volume was 10 μL, and the detection wavelength was set at 254 nm. Gradient elution was carried out as follows: 0–15 min, 95% → 91% A; 15–20 min, 91% → 80% A; 20–23 min, 80% → 79% A; 23–30 min, 79% → 35% A; and 30–35 min, 35% → 35% A.

### 4.5. Methodology Validation

#### 4.5.1. Repeatability

The same sample was extracted five times, and each extract was injected with 10 μL into the HPLC system. The retention times and content percentages of each iridoid glycoside were calculated, and the RSD values were used to evaluate the method’s repeatability.

#### 4.5.2. Investigation of Linear Relationship

The mixed standard stock solution was diluted to a series of concentrations and analyzed by HPLC. Linear regression was performed with the standard concentrations as the x-axis and the corresponding peak areas as the y-axis.

#### 4.5.3. Precision

The same sample was consecutively injected five times, and the retention times and content percentages of each target compound were calculated to determine the RSD.

#### 4.5.4. Stability

The same sample was injected (10 μL) at 0, 2, 8, 12, and 24 h after extraction. The retention times and content percentages of each target compound were calculated, and the RSD values were used to evaluate the method’s stability.

#### 4.5.5. Recovery Rate

Before HPLC analysis, a suitable amount of the powdered sample was spiked with the mixed standard solution and processed in accordance with the procedure described in Section 4.3.1. We determined the retention time and content of each target compound, and then calculated their recovery and RSD values. Here is one example of Equation (1):

(1) $$\mathrm{Recovery}\;\mathrm{rate}\;(\%)=\frac{\mathrm{Measured}\;\mathrm{content}\;\mathrm{of}\;\mathrm{spiked}\;\mathrm{sample}\;-\;\mathrm{Native}\;\mathrm{content}\;\mathrm{of}\;\mathrm{sample}\;}{\mathrm{Amount}\;\mathrm{of}\;\mathrm{standard}\;\mathrm{added}}\times 100$$

#### 4.5.6. LOD

The standard solution was injected into the HPLC system at volumes of 0.1 μL, 0.2 μL, 0.3 μL, 0.4 μL, and 0.5 μL. LOD was defined as the concentration corresponding to a S/N of 3:1.

### 4.6. Antioxidant Activity Evaluation Methods

#### 4.6.1. Assay of DPPH Radical Scavenging Activity

The assay was conducted based on the method of Rumpf et al. [61] with slight modifications. A Trolox standard curve was established, and the samples were diluted tenfold prior to the analysis. Three groups were arranged in a 96-well plate: a control group (400 μL of sample solution + 600 μL of 80% methanol), a test group (400 μL of sample solution + 600 μL of DPPH working solution), and a blank group (600 μL of DPPH working solution + 400 μL of 80% methanol). After the incubation at room temperature in the dark for 30 min, the absorbance of each well was measured at 517 nm. All measurements were performed in triplicate, and the results were expressed as the mean values. The radical scavenging activity was calculated according to the following Equation (2):

(2) $$\mathrm{DPPH}\;\mathrm{radical}\;\mathrm{scavenging}\;\mathrm{activity}\;(\%)\;=\;\left(1-\frac{A_{\mathrm{sample}}-A_{\mathrm{control}}}{A_{\mathrm{blank}}}\right)\times 100$$

The sample’s equivalent Trolox concentration was determined from the standard curve after calculating the DPPH radical scavenging activity. TEAC was calculated according to the following Equation (3):

(3) $$\mathrm{TEAC}=\frac{C_{\mathrm{sample}}\times V_{\mathrm{total}}\times D}{m\times M}$$

In this equation, *C*_sample_ is the Trolox concentration (μg·mL^−1^) corresponding to the sample on the standard curve, *V*_total_ refer to the total volume of the sample solution participating in the reaction (mL), *D* is the dilution factor of the sample, *m* means the mass of the sample (g), and M refers to the molar mass of Trolox, taken as 250.29 μg·μmol^−1^.

TEAC values were expressed as μmol TE·g^−1^ (DW) of dry sample, reflecting the samples’ antioxidant capacity. Higher values of TEAC indicate stronger free radical scavenging activity and, consequently, greater antioxidant potential.

#### 4.6.2. Assay of ABTS Radical Scavenging Activity

The assay was performed with slight modifications based on the method proposed by Re et al. [62]. A Trolox standard curve was established, and ABTS working solution was prepared according to the manufacturer’s instructions and stored at room temperature in the dark. A mixture of 100 μL of the original sample solution, 170 μL of ABTS working solution, and 20 μL of enzyme solution was prepared, incubated at room temperature in the dark for 6 min, and the absorbance of each mixture was measured at 405 nm. All measurements were performed in triplicate, and the results were expressed as the mean values. The antioxidant capacity was expressed as TEAC. Here is one example of Equation (4):

(4) $$\mathrm{TEAC}=\frac{C_{\mathrm{sample}}\times V_{\mathrm{total}}\times D}{m}$$

In this equation, *C*_sample_ refers to the Trolox concentration (μmol·mL^−1^) corresponding to the sample on the standard curve. TEAC values were expressed as μmol TE·g^−1^ (DW) of dry sample.

#### 4.6.3. Assay of FRAP

The assay was conducted with slight modifications based on the method of Wang et al. [63]. FRAP working solution was prepared according to the manufacturer’s instructions at a specified ratio, and the FeSO_4_·7H_2_O standard was diluted to generate a series of concentrations for the standard curve. The samples were diluted twofold, and 180 μL of FRAP working solution was added to a 96-well plate along with 5 μL of sample solution or methanol (blank). The plate was incubated at 37 °C for 3–5 min, and the optical density (OD) of each well was measured at 593 nm using a microplate reader. All measurements were performed in triplicate, and the mean values were recorded. The OD values of the samples, corrected by subtracting the blank OD, were employed in the standard curve to determine the FeSO_4_ equivalents, which were then used to calculate FRAP values according to the following Equation (5):

(5) $$\mathrm{FRAP}=\frac{C_{{\mathrm{Fe}}^{2+}}\times V_{\mathrm{total}}\times D}{m}$$

In this equation, *C*_Fe_^2+^ refers to the concentration of Fe^2+^ (μmol·mL^−1^).

The results were expressed as samples’ μmol Fe^2+^·g^−1^ (DW). Higher FRAP values indicate stronger reducing antioxidant capacity, reflecting samples’ greater electron-donating ability and potential antioxidant activity.

### 4.7. Data Processing

The membership function is a widely employed tool in fuzzy mathematical analysis and has been extensively applied for the comprehensive evaluation of crop quality, stress tolerance, and medicinal material quality [43]. To achieve a multidimensional assessment of *G. siphonantha* quality, the membership function values (*M_ij_*) of each iridoid glycoside were calculated for every sample based on the contents of five iridoid glycosides. The comprehensive membership function value (*M_i_*) for each sample was analyzed accordingly [64]. Here are Equations (6) and (7):

(6) $$M_{ij}=\frac{X_{ij}-X_{j\min}}{X_{j\max}-X_{j\min}}$$

(7) $$M_{i}=\frac{1}{n}\sum_{j=1}^{n}M_{ij}$$

Here, *i* represents the sample group index, and *j* = 1, 2, …, 5 corresponds to the five iridoid glycosides: gentiopicroside, loganic acid, sweroside, swertiamarin, and 6′-O-β-D-glucosylgentiopicroside. *X_ij_* denotes the content of the *j*th compound in the *i*th sample group, while *X_j_*_min_ and *X_j_*_max_ represent the minimum and maximum contents of the *j*th compound, respectively.

The entropy weight–TOPSIS method integrates the objective weighting mechanism of the entropy weight method with the distance-based evaluation principle of TOPSIS. The raw data are first standardized, and the *E_j_*, *D_j_*, and *W_j_* are calculated based on the entropy theory. The standardized data are then multiplied by the corresponding weights to obtain the weighted matrix, from which the *D_i_*^+^ and *D_i_*^−^ are constructed. By calculating the Euclidean distances between each sample and the positive and negative ideal solutions, the *C_i_* is finally obtained to represent the degree to which each sample approaches the optimal solution; a higher *C_i_* value indicates better overall performance [65]. This method ensures objective weight determination while effectively assessing the relative advantages of samples, making it suitable for multi-indicator comprehensive evaluation. Here are Equations (8) and (9):

(8) $$W_{j}=\frac{1-E_{j}}{\sum_{j=1}^{m}\left(1-E_{j}\right)}$$

(9) $$C_{i}=\frac{D_{i}^{-}}{D_{i}^{+}+D_{i}^{-}}$$

ANOVA for the active compounds was performed using R version 4.5.0, followed by Tukey’s HSD test for multiple pairwise comparisons. Differences were considered statistically significant at *p* < 0.05. For comprehensive quality assessment, PCA and Spearman’s rank correlation analysis were performed using Origin 2021 (64-bit), OPLS-DA was conducted with SIMCA 14.1, and GRA was carried out via the SPSSAU online platform (https://spssau.com, accessed 13 January 2026).

## 5. Conclusions

Through a systematic investigation of the accumulation patterns of iridoid glycosides in *G. siphonantha* and their associated antioxidant activities, we find that 3-year-old cultivated samples exhibited the highest total content of the five iridoid glycosides, and we also find that cultivated samples demonstrated greater quality stability compared with wild resources. Among the cultivated samples harvested in the same month, the 3-year cultivation stage was identified as the optimal harvest age, whereas June–July of the fourth cultivation year was found to be the most suitable harvest period. The results of PCA, OPLS-DA, membership function analysis, and entropy weight–TOPSIS analysis further validated the accuracy of these findings. Regarding antioxidant properties, the antioxidant activity of cultivated materials increased progressively from two to four years of growth, with stronger activities generally observed in June and August. GRA and Spearman’s rank correlation analysis demonstrated positive correlations between the total iridoid glycoside contents and all three antioxidant indices, suggesting that iridoid glycosides may serve as one of the key material bases responsible for the antioxidant effects. This study provides valuable references for *G. siphonantha*’s quality control and industrial development, and lays a scientific foundation for the sustainable utilization of *Gentiana* resources.

## Figures and Tables

**Table 1:** Linear regression equations of five iridoid glycosides.

Component	Linear Regression Equation	*R* ^2^	Linearity Range/(mg·mL^−1^)	LOD/ng
Gentiopicroside	*Y* = 8416.1 *X* − 0.3633	0.9999	0.0375–11.25	2.00
Loganic acid	*Y* = 7657.5 *X* + 0.3352	0.9999	0.0015–0.45	2.25
Sweroside	*Y* = 15,009 *X* + 0.2432	0.9999	0.0021–0.63	0.50
Swertiamarin	*Y* = 9023.2 *X* + 0.1280	1.0000	0.0025–0.75	2.25
6′-O-β-D-glucosylgentiopicroside	*Y* = 6511.4 *X* + 0.1083	1.0000	0.0026–0.78	2.00

**Table 2:** The results of precision, repeatability, stability and recovery rate tests.

Types of Compounds	Methodology Validation	Precision	Repeatability	Stability	Recovery Rate
Gentiopicroside	Content RSD (%)	0.98	0.59	0.26	0.17
	Retention time RSD (%)	0.88	0.39	0.95	0.19
	Recovery rate (%)				103.64
Loganic acid	Content RSD (%)	1.47	1.58	0.55	0.35
	Retention time RSD (%)	0.76	0.47	1.32	0.19
	Recovery rate (%)				109.59
Sweroside	Content RSD (%)	0.46	0.99	0.68	0.18
	Retention time RSD (%)	0.94	0.34	0.69	0.15
	Recovery rate (%)				105.02
Swertiamarin	Content RSD (%)	1.25	0.89	0.83	0.06
	Retention time RSD (%)	0.99	0.48	0.88	0.18
	Recovery rate (%)				99.15
6′-O-β-D-glucosylgentiopicroside	Content RSD (%)	0.64	0.81	0.51	0.19
	Retention time RSD (%)	0.64	0.51	0.80	0.19
	Recovery rate (%)				100.77

**Table 3:** Membership function calculation results of five iridoid glycoside components from different sources.

Sample Source	Sample Information	Membership Function Value					
		Gentiopicroside	Loganic Acid	Sweroside	Swertiamarin	6′-O-β-D- Glucosylgentiopicroside	Overall Membership Function Value
Cultivated	2-year-old	0.437	0.008	0.344	0.392	0.347	0.306
	3-year-old	0.959	0.615	1.000	0.936	1.000	0.902
	4-year-old	0.973	0.001	0.062	1.000	0.554	0.518
Wild	Sample 1	1.000	1.000	0.709	0.997	0.287	0.799
	Sample 2	0.216	0.630	0.214	0.231	0.159	0.290
	Sample 3	0.514	0.705	0.954	0.590	0.427	0.638
	Sample 4	0.000	0.000	0.000	0.000	0.000	0.000
	Sample 5	0.360	0.375	0.140	0.390	0.262	0.305

**Table 4:** Membership function calculation results of five iridoid glycoside components in cultivated materials from different months.

Collection Month	Membership Function Value					
	Gentiopicroside	Loganic Acid	Sweroside	Swertiamarin	6′-O-β-D- Glucosylgentiopicroside	Overall Membership Function Value
4	0.000	1.000	0.276	0.040	0.553	0.374
5	0.206	0.087	0.079	0.475	0.171	0.204
6	1.000	0.000	0.327	1.000	0.357	0.537
7	0.953	0.315	1.000	0.731	0.000	0.600
8	0.667	0.093	0.118	0.478	0.086	0.289
9	0.566	0.220	0.050	0.000	0.398	0.247
10	0.015	0.294	0.000	0.084	1.000	0.279

Notes: *E_j_*, information entropy of the *j*-th indicator; *D_j_*, information utility value (degree of diversification) reflecting the discriminative power of the *j*-th indicator; *W_j_*, weight of the *j*-th indicator.

Notes: *D_i_*^+^, Euclidean distance of each sample to the positive ideal solution; *D_i_*^−^, Euclidean distance of each sample to the negative ideal solution; *C_i_*, closeness coefficient, indicating the relative proximity of each sample to the ideal solution.

Notes: *E_j_*, information entropy of the *j*-th indicator; *D_j_*, information utility value (degree of diversification) reflecting the discriminative power of the *j*-th indicator; *W_j_*, weight of the *j*-th indicator.

Notes: *D_i_*^+^, Euclidean distance of each sample to the positive ideal solution; *D_i_*^−^, Euclidean distance of each sample to the negative ideal solution; *C_i_*, closeness coefficient, indicating the relative proximity of each sample to the ideal solution.

**Table 5:** Results of *E_j_*, *D_j_*, and *W_j_* for *G. siphonantha* samples from different sources.

Component	*E_j_*	*D_j_*	*W_j_*
Gentiopicroside	0.878	0.122	0.148
Loganic acid	0.762	0.238	0.290
Sweroside	0.795	0.205	0.250
Swertiamarin	0.882	0.118	0.144
6′-O-β-D-glucosylgentiopicroside	0.862	0.138	0.168

Notes: *E_j_*, information entropy of the *j*-th indicator; *D_j_*, information utility value (degree of diversification) reflecting the discriminative power of the *j*-th indicator; *W_j_*, weight of the *j*-th indicator.

**Table 6:** Relative correlation degree and ranking of cultivated *G. siphonantha* in different sources.

Sample Source	Sample Information	*D_i_* ^+^	*D_i_* ^−^	*C_i_*
Cultivated	2-year-old	0.369	0.135	0.267
	3-year-old	0.112	0.401	0.782
	4-year-old	0.380	0.225	0.371
Wild	Sample 1	0.140	0.400	0.740
	Sample 2	0.310	0.197	0.389
	Sample 3	0.159	0.342	0.682
	Sample 4	0.466	0.000	0.000
	Sample 5	0.333	0.145	0.303

Notes: *D_i_*^+^, Euclidean distance of each sample to the positive ideal solution; *D_i_*^−^, Euclidean distance of each sample to the negative ideal solution; *C_i_*, closeness coefficient, indicating the relative proximity of each sample to the ideal solution.

**Table 7:** Results of *E_j_*, *D_j_*, and *W_j_* for *G. siphonantha* samples from different months.

Component	*E_j_*	*D_j_*	*W_j_*
Gentiopicroside	0.787	0.213	0.213
Loganic acid	0.743	0.257	0.257
Sweroside	0.687	0.313	0.313
Swertiamarin	0.766	0.234	0.234
6′-O-β-D-glucosylgentiopicroside	0.802	0.198	0.198

Notes: *E_j_*, information entropy of the *j*-th indicator; *D_j_*, information utility value (degree of diversification) reflecting the discriminative power of the *j*-th indicator; *W_j_*, weight of the *j*-th indicator.

**Table 8:** Relative correlation degree and ranking of cultivated *G. siphonantha* in different months.

Collection Month	*D_i_* ^+^	*D_i_* ^−^	*C_i_*	Rank
4	0.324	0.241	0.426	3
5	0.376	0.106	0.220	7
6	0.293	0.280	0.488	2
7	0.224	0.344	0.605	1
8	0.352	0.154	0.304	5
9	0.374	0.128	0.255	6
10	0.387	0.175	0.312	4

Notes: *D_i_*^+^, Euclidean distance of each sample to the positive ideal solution; *D_i_*^−^, Euclidean distance of each sample to the negative ideal solution; *C_i_*, closeness coefficient, indicating the relative proximity of each sample to the ideal solution.

**Table 9:** Gray relational grades between five iridoid glycosides, total iridoid glycoside content, and antioxidant activities.

No.	DPPH Radical Scavenging Activity		ABTS Radical Scavenging Activity		FRAP Radical Scavenging Activity	
	Component	*γ*	Component	*γ*	Component	*γ*
1	Gentiopicroside	0.890	Swertiamarin	0.914	Swertiamarin	0.894
2	Total content of iridoid glycosides	0.889	Total content of iridoid glycosides	0.901	Gentiopicroside	0.861
3	Swertiamarin	0.878	Gentiopicroside	0.898	Total content of iridoid glycosides	0.852
4	6′-O-β-D- glucosylgentiopicroside	0.802	6′-O-β-D- glucosylgentiopicroside	0.763	6′-O-β-D- glucosylgentiopicroside	0.732
5	Loganic acid	0.685	Loganic acid	0.699	Loganic acid	0.678
6	Sweroside	0.579	Sweroside	0.571	Sweroside	0.541

**Table 10:** Sample information table of *G. siphonantha*.

Sample No.	Sample Type	Collection Time	Collection Location	Latitude	Longitude	Altitude (m)
1	2-year-old cultivated	August 2021	Huangzhong District, Xining City, Qinghai Province	36.4434° N	101.5136° E	2901.23
2	3-year-old cultivated	August 2022				
3	4-year-old cultivated	April 2023				
4	4-year-old cultivated	May 2023				
5	4-year-old cultivated	June 2023				
6	4-year-old cultivated	July 2023				
7	4-year-old cultivated	August 2023				
8	4-year-old cultivated	September 2023				
9	4-year-old cultivated	October 2023				
10	wild sample 1	August 2023	Datong County, Xining City, Qinghai Province	37.1318° N	101.5057° E	2812.35
11	wild sample 2	August 2023	Delingha City, Haixi Prefecture, Qinghai Province	37.4946° N	97.4587° E	3850.06
12	wild sample 3	August 2023	Tianjun County, Haixi Prefecture, Qinghai Province	37.4286° N	98.3611° E	3798.31
13	wild sample 4	August 2023	Tianjun County, Haixi Prefecture, Qinghai Province	37.1771° N	98.8681° E	3861.63
14	wild sample 5	August 2023	Qilian County, Haibei Prefecture, Qinghai Province	37.9286° N	100.1483° E	3603.38

## Data Availability

The data that support the findings of this study are available from the corresponding author upon reasonable request.

## Supplementary Materials

The following supporting information can be downloaded at: https://www.mdpi.com/article/10.3390/molecules31020312/s1. Table S1: The results of precision, repeatability, stability and standard addition recovery tests. Table S2: ANOVA analysis of individual and total iridoid glycoside contents of iridoid glycosides in *G. siphonantha* collected in different wild locations. Table S3: The results for the contents of individual secoiridoid compounds and their total amount in the investigated *G. siphonantha* collected in different wild locations. Table S4: ANOVA analysis of individual and total iridoid glycoside contents of iridoid glycosides in *G. siphonantha* collected at different ages. Table S5: The results for individual and total iridoid glycoside contents in the investigated *G. siphonantha* collected at different ages. Table S6: ANOVA analysis of individual and total iridoid glycoside contents in *G. siphonantha* collected in different months. Table S7: The results for the contents of individual iridoid compounds and their total amount in the investigated *G. siphonantha* collected in different months. Table S8: Measured physicochemical properties of soil at the cultivation location.

## Abbreviations

The following abbreviations are used in this manuscript:

ABTS	2,2′-azino-bis (3-ethylbenzothiazoline-6-sulfonic acid)
ANOVA	Analysis of variance
*C_i_*	Closeness coefficient
ChP	Pharmacopoeia of the People’s Republic of China
DW	Dry weight
*D_i_*^+^	Euclidean distance of each sample to the positive ideal solution
*D_i_*^−^	Euclidean distance of each sample to the negative ideal solution
*D_j_*	Information utility value
DPPH	1,1-diphenyl-2-picrylhydrazyl
*E_j_*	Information entropy
FRAP	Ferric reducing antioxidant power
GRA	Gray relation analysis
HPLC	High-performance liquid chromatography
LOD	Limit of detection
OD	Optical density
OPLS-DA	Orthogonal partial least squares–discriminant analysis
PCA	Principal component analysis
RSD	Relative standard deviation
S/N	Signal-to-noise ratio
TEAC	Trolox equivalent antioxidant capacity
VIP	Variable importance in projection
*W_j_*	indicator weights
*γ*	Gray relational coefficients
