# Supplementary material for: Quality Evaluation and Antioxidant Activity of Cultivated Gentiana siphonantha: An Ethnic Medicine from the Tibetan Plateau
Source: Molecules. 2026 Jan 16;31(2):312. doi: 10.3390/molecules31020312 (PMC12844228; doi:10.3390/molecules31020312)
Supplement: Supplementary file 1 [file molecules-31-00312-s001.zip › molecules-4069187-supplementary.pdf]

Table S1. The results of precision, repeatability, stability and standard addition recovery tests.

	**Precision**				**Repeatability**				**Stability**				**Recovery rate**				
	**Content（$mg·g^{-1}$）**	**Content RSD(%)**	**Retention time（min）**	**Retention time RSD(%)**	**Content（$mg·g^{-1}$）**	**Content RSD(%)**	**Retention time（min）**	**Retention time RSD(%)**	**Content（$mg·g^{-1}$）**	**Content RSD(%)**	**Retention time（min）**	**Retention time RSD(%)**	**Content（$mg·g^{-1}$）**	**Content RSD(%)**	**Retention time（min）**	**Retention time RSD(%)**	**Recovery rate (%)**
Gentiopicroside	120.35		18.56		123.50		18.32		122.17		18.60		129.80		18.10		
	121.03		18.57		122.25		18.28		122.30		18.50		130.20	0.17	18.11	0.19	103.64
	120.77	0.98	18.23	0.88	122.12	0.59	18.17	0.39	122.65	0.26	18.48	0.95	129.84		18.17		
	119.14		18.56		122.47		18.18		122.73		18.37						
	122.42		18.31		123.66		18.17		122.94		18.14						
Loganic acid	1.04		11.77		1.06		11.54		0.99		11.82		1.32		11.44		
	1.00		11.75		1.02		11.52		0.99		11.71		1.33	0.35	11.43	0.19	109.59
	1.03	1.47	11.61	0.76	1.02	1.58	11.43	0.47	0.99	0.55	11.67	1.32	1.32		11.47		
	1.01		11.74		1.04		11.43		0.99		11.62						
	1.02		11.58		1.05		11.44		0.98		11.41						
Sweroside	1.08		19.97		1.10		19.81		1.10		20.00		1.31		19.61		
	1.07		19.98		1.09		19.78		1.10		19.93		1.31	0.18	19.63	0.15	105.02
	1.08	0.46	19.64	0.94	1.10	0.99	19.67	0.34	1.10	0.68	19.91	0.69	1.31		19.66		
	1.08		19.96		1.11		19.69		1.10		19.82						
	1.08		19.62		1.12		19.67		1.11		19.65						
Swertiamarin	3.14		15.49		3.17		15.26		3.23		15.54		3.53		15.06		
	3.09		15.50		3.21		15.23		3.16		15.45		3.53	0.06	15.07	0.18	99.15
	3.19	1.25	15.22	0.99	3.22	0.89	15.10	0.48	3.19	0.83	15.41	0.88	3.52		15.11		
	3.11		15.48		3.25		15.12		3.18		15.32						
	3.14		15.21		3.24		15.12		3.20		15.18						
6'-O-β-D-glucosylgentiopicroside	1.79		16.25		1.88		15.98		1.86		16.32		2.07		15.76		
	1.80		16.26		1.88		15.95		1.84		16.22		2.06	0.19	15.78	0.19	100.77
	1.79	0.64	16.07	0.64	1.84	0.81	15.81	0.51	1.85	0.51	16.16	0.80	2.06		15.82		
	1.80		16.24		1.87		15.83		1.85		16.05						
	1.77		16.06		1.86		15.83		1.85		16.38						

Note: All results are expressed as $mg·g^{-1}$ DM.

Table S2. ANOVA analysis of individual and total iridoid glycoside contents of iridoid glycosides in *G. siphonantha* collected in different wild locations.

	Variation source	Sum of squares	*df*	Mean square	*F*	Significance
Gentiopicroside	Treatment	17335.778	4	4333.945	39461.870	***
	Residual	1.098	10	0.110		
	Total	17336.877	14			
Loganic acid	Treatment	32.207	4	8.052	3393.036	***
	Residual	0.024	10	0.002		
	Total	32.230	14			
Sweroside	Treatment	6.381	4	1.595	5899.652	***
	Residual	0.003	10	0.000		
	Total	6.384	14			
Swertiamarin	Treatment	8.320	4	2.080	2851.785	***
	Residual	0.007	10	0.001		
	Total	8.327	14			
6'-O-β-D-glucosylgentiopicroside	Treatment	0.969	4	0.242	392.599	***
	Residual	0.006	10	0.001		
	Total	0.975	14			
Total content of iridoid glycosides	Treatment	20206.278	4	5051.569	37431.695	***
	Residual	1.350	10	0.135		
	Total	20207.627	14			

Note: *$p < 0.05$; **$p < 0.01$; ***$p < 0.001$.

Table S3. The contents of individual iridoid compounds and their total amount in the investigated *G. siphonantha* from different wild locations, along with Tukey's HSD test results.

	Gentiopicroside		Loganic acid		Sweroside		Swertiamarin		6'-O-β-D-glucosylgentiopicroside		Total content of iridoid glycosides	
	**Replicate ($mg{\cdot}g^{-1}$)**	**Content ($mg{\cdot}g^{-1}$)**	**Replicate ($mg{\cdot}g^{-1}$)**	**Content ($mg{\cdot}g^{-1}$)**	**Replicate ($mg{\cdot}g^{-1}$)**	**Content ($mg{\cdot}g^{-1}$)**	**Replicate ($mg{\cdot}g^{-1}$)**	**Content ($mg{\cdot}g^{-1}$)**	**Replicate ($mg{\cdot}g^{-1}$)**	**Content ($mg{\cdot}g^{-1}$)**	**Replicate ($mg{\cdot}g^{-1}$)**	**Content ($mg{\cdot}g^{-1}$)**
	127.950		5.847		1.504		3.028		1.060		139.388	
Wild sample 1	126.931	127.330 ± 0.544 a	5.841	5.849 ± 0.009 a	1.506	1.504 ± 0.002 b	3.037	3.018 ± 0.025 a	1.090	1.075 ± 0.015 b	138.405	138.776 ± 0.534 a
	127.109		5.859		1.502		2.989		1.076		138.535	
	48.501		4.289		0.628		1.362		0.892		55.672	
Wild sample 2	47.859	48.171 ± 0.321 d	4.215	4.236 ± 0.046 c	0.608	0.618 ± 0.010 c	1.310	1.332 ± 0.027 d	0.816	0.847 ± 0.040 c	54.808	55.204 ± 0.437 d
	48.152		4.203		0.617		1.325		0.834		55.132	
	78.262		4.558		1.941		2.132		1.337		88.230	
Wild sample 3	78.338	78.291 ± 0.041 b	4.648	4.563 ± 0.082 b	1.976	1.941 ± 0.035 a	2.149	2.122 ± 0.033 b	1.348	1.326 ± 0.028 a	88.459	88.244 ± 0.208 b
	78.274		4.485		1.907		2.085		1.295		88.044	
	26.301		1.464		0.235		0.818		0.556		29.375	
Wild sample 4	26.515	26.374 ± 0.122 e	1.519	1.493 ± 0.028 e	0.234	0.235 ± 0.001 e	0.818	0.824 ± 0.010 e	0.553	0.563 ± 0.015 d	29.639	29.489 ± 0.136 e
	26.306		1.496		0.236		0.835		0.579		29.452	
	62.477		3.105		0.481		1.643		1.012		68.718	
Wild sample 5	62.458	62.678 ± 0.365 c	3.178	3.125 ± 0.046 d	0.481	0.485 ± 0.008 d	1.699	1.681 ± 0.033 c	1.046	1.031 ± 0.018 b	68.862	69.000 ± 0.371 c
	63.099		3.091		0.494		1.701		1.034		69.420	

Notes: All results are expressed as $mg{\cdot}g^{-1}$ DM. Content values are expressed as mean ± SD (n = 3). Values in the same column followed by different lowercase letters indicate significant differences at $p < 0.05$ (Tukey's HSD test).

Table S4. ANOVA analysis of individual and total iridoid glycoside contents of iridoid glycosides in *G. siphonantha* collected in different ages.

	**Variation source**	**Sum of squares**	***df***	**Mean square**	***F***	**Significance**
Gentiopicroside	Treatment	5710.833	2	2855.417	1134.530	***
	Residual	15.101	6	2.517		
	Total	5725.934	8			
Loganic acid	Treatment	14.147	2	7.074	6176.644	***
	Residual	0.007	6	0.001		
	Total	14.154	8			
Sweroside	Treatment	4.450	2	2.225	12202.589	***
	Residual	0.001	6	0.000		
	Total	4.451	8			
Swertiamarin	Treatment	3.244	2	1.622	2182.798	***
	Residual	.004	6	0.001		
	Total	3.248	8			
6'-O-β-D-glucosylgentiopicroside	Treatment	2.132	2	1.066	555.153	***
	Residual	0.012	6	0.002		
	Total	2.144	8			
Total content of iridoid glycosides	Treatment	6540.132	2	3270.066	1157.577	***
	Residual	16.950	6	2.825		
	Total	6557.082	8			

Note: *$p < 0.05$; **$p < 0.01$; ***$p < 0.001$.

Table S5. The contents of individual iridoid compounds and their total amount in the investigated *G. siphonantha* from different ages, along with Tukey's HSD test results.

	**Gentiopicroside**		**Loganic acid**		**Sweroside**		**Swertiamarin**		**6'-O-β-D-glucosylgentiopicroside**		**Total content of iridoid glycosides**	
	**Replicate ($mg \cdot g^{-1}$)**	**Content ($mg \cdot g^{-1}$)**	**Replicate ($mg \cdot g^{-1}$)**	**Content ($mg \cdot g^{-1}$)**	**Replicate ($mg \cdot g^{-1}$)**	**Content ($mg \cdot g^{-1}$)**	**Replicate ($mg \cdot g^{-1}$)**	**Content ($mg \cdot g^{-1}$)**	**Replicate ($mg \cdot g^{-1}$)**	**Content ($mg \cdot g^{-1}$)**	**Replicate ($mg \cdot g^{-1}$)**	**Content ($mg \cdot g^{-1}$)**
	70.668		1.553		0.849		1.702		1.197		75.969	
2-old-year sample	70.482	70.457 ± 0.224 b	1.514	1.528 ± 0.022 b	0.850	0.850 ± 0.001 b	1.689	1.686 ± 0.017 c	1.188	1.183 ± 0.016 c	75.724	75.705 ± 0.274 b
	70.222		1.517		0.849		1.668		1.166		75.422	
	121.248		4.131		2.004		2.845		2.268		132.496	
3-old-year sample	125.644	123.171 ± 2.249 a	4.234	4.172 ± 0.055 a	2.049	2.024 ± 0.023 a	2.929	2.884 ± 0.042 b	2.400	2.350 ± 0.072 a	137.256	134.601 ± 2.427 a
	122.620		4.152		2.018		2.877		2.383		134.050	
	122.894		1.496		0.343		3.029		1.533		129.294	
4-old-year sample	125.973	124.588 ± 1.562 a	1.498	1.498 ± 0.002 b	0.343	0.349 ± 0.004 c	3.033	3.024 ± 0.011 a	1.569	1.554 ± 0.019 b	132.416	131.009 ± 1.584 a
	124.897		1.499		0.350		3.011		1.560		131.317	

Notes: All results are expressed as $mg \cdot g^{-1}$ DM. Content values are expressed as mean ± SD (n = 3). Values in the same column followed by different lowercase letters indicate significant differences at $p < 0.05$ (Tukey's HSD test).

Table S6. ANOVA analysis of individual and total iridoid glycoside contents in *G. siphonantha* collected in different months.

	**Variation source**	**Sum of squares**	***df***	**Mean square**	***F***	**Significance**
Gentiopicroside	Treatment	3142.936	6	523.823	719.881	***
	Residual	10.187	14	0.728		
	Total	3153.123	20			
Loganic acid	Treatment	43.668	6	7.278	5082.950	***
	Residual	0.020	14	0.001		
	Total	43.688	20			
Sweroside	Treatment	7.198	6	1.200	7778.165	***
	Residual	0.002	14	0.000		
	Total	7.200	20			
Swertiamarin	Treatment	2.224	6	0.371	4.579	**
	Residual	1.133	14	0.081		
	Total	3.357	20			
6'-O-β-D-glucosylgentiopicroside	Treatment	7.750	6	1.292	895.574	***
	Residual	0.020	14	0.001		
	Total	7.770	20			
Total content of iridoid glycosides	Treatment	2889.042	6	481.507	530.877	***
	Residual	12.698	14	0.907		
	Total	2901.741	20			

Note: *$p < 0.05$; **$p < 0.01$; ***$p < 0.001$.

Table S7. The contents of individual iridoid compounds and their total amount in the investigated *G. siphonantha* from different months, along with Tukey's HSD test results.

	**Gentiopicroside**		**Loganic acid**		**Sweroside**		**Swertiamarin**		**6'-O-β-D-glucosylgentiopicroside**		**Total content of iridoid glycosides**	
	**Replicate ($mg·g^{-1}$)**	**Content ($mg·g^{-1}$)**	**Replicate ($mg·g^{-1}$)**	**Content ($mg·g^{-1}$)**	**Replicate ($mg·g^{-1}$)**	**Content ($mg·g^{-1}$)**	**Replicate ($mg·g^{-1}$)**	**Content ($mg·g^{-1}$)**	**Replicate ($mg·g^{-1}$)**	**Content ($mg·g^{-1}$)**	**Replicate ($mg·g^{-1}$)**	**Content ($mg·g^{-1}$)**
April sample	102.851	103.587 ± 0.768 e	5.647	5.713 ± 0.057 a	0.635	0.636 ± 0.004 c	2.618	2.620 ± 0.004 b	2.475	2.458 ± 0.015 b	114.227	115.014 ± 0.809 cd
	104.383		5.752		0.633		2.624		2.452		115.843	
	103.526		5.740		0.640		2.617		2.447		114.970	
May sample	110.036	110.076 ± 0.176 d	1.461	1.469 ± 0.013 d	0.273	0.274 ± 0.001 e	3.038	3.021 ± 0.018 ab	1.703	1.719 ± 0.014 d	116.510	116.559 ± 0.181 c
	109.923		1.483		0.274		3.003		1.724		116.408	
	110.269		1.462		0.275		3.024		1.730		116.759	
June sample	135.320	135.059 ± 0.624 a	1.061	1.064 ± 0.004 e	0.729	0.728 ± 0.001 b	3.513	3.506 ± 0.018 a	2.083	2.078 ± 0.022 c	142.706	142.435 ± 0.594 a
	134.346		1.064		0.728		3.519		2.097		141.755	
	135.510		1.068		0.728		3.485		2.055		142.845	
July sample	133.069	133.588 ± 1.265 a	2.563	2.527 ± 0.080 b	1.970	1.959 ± 0.032 a	3.326	3.257 ± 0.110 ab	1.431	1.387 ± 0.065 f	142.360	142.718 ± 1.465 a
	132.666		2.435		1.923		3.130		1.312		141.466	
	135.030		2.581		1.985		3.315		1.418		144.329	
August sample	122.894	124.588 ± 1.562 b	1.496	1.498 ± 0.002 d	0.343	0.349 ± 0.004 d	3.029	3.024 ± 0.011 ab	1.533	1.554 ± 0.019 e	129.294	131.009 ± 1.584 b
	125.973		1.498		0.343		3.033		1.569		132.416	
	124.897		1.499		0.350		3.011		1.560		131.317	
September sample	121.514	121.408 ± 0.092 c	2.072	2.087 ± 0.014 c	0.222	0.221 ± 0.001 f	2.998	2.583 ± 0.744 b	2.154	2.158 ± 0.010 c	128.959	128.458 ± 0.786 b
	121.363		2.093		0.221		1.724		2.150		127.551	
	121.348		2.097		0.221		3.027		2.169		128.862	
October sample	104.096	104.074 ± 0.183 e	2.426	2.429 ± 0.006 b	0.132	0.130 ± 0.001 g	2.669	2.660 ± 0.007 b	3.401	3.325 ± 0.067 a	112.723	112.619 ± 0.196 d
	103.882		2.426		0.130		2.657		3.298		112.393	
	104.246		2.436		0.129		2.655		3.275		112.741	

Notes: All results are expressed as $mg·g^{-1}$ DM. Content values are expressed as mean ± SD (n = 3). Values in the same column followed by different lowercase letters indicate significant differences at $p < 0.05$ (Tukey's HSD test).

Table S8. Measured physicochemical properties of soil at the cultivation location.

Soil property	Measured value	Safety limit (HJ 804-2016, pH > 7.5)
pH	8.20	
Total N ($g \cdot kg^{-1}$, dry weight)	0.39	
Total P ($g \cdot kg^{-1}$, dry weight)	0.68	
Total K ($g \cdot kg^{-1}$, dry weight)	17.10	
Alkali-hydrolyzable N ($mg \cdot kg^{-1}$)	22.00	
Available P ($mg \cdot kg^{-1}$)	14.4	
Available K ($mg \cdot kg^{-1}$, dry weight)	222.00	
Organic matter ($g \cdot kg^{-1}$, dry weight)	6.04	
Total salt content ($g \cdot kg^{-1}$)	1.20	
C/N ratio	8.98	
Pb ($mg \cdot kg^{-1}$, dry weight)	3.68	< 170
Cd ($mg \cdot kg^{-1}$, dry weight)	0.06	< 0.6
Hg ($mg \cdot kg^{-1}$, dry weight)	0.05	< 3.4
As ($mg \cdot kg^{-1}$, dry weight)	20.60	< 25
